# Insights into the binding mode of AS1411 aptamer to nucleolin

**DOI:** 10.3389/fmolb.2022.1025313

**Published:** 2022-10-03

**Authors:** Lihua Bie, Yue Wang, Fuze Jiang, Zhen Xiao, Lianjun Zhang, Jing Wang

**Affiliations:** ^1^ Hubei Key Laboratory of Agricultural Bioinformatics, College of Informatics, Huazhong Agricultural University, Wuhan, China; ^2^ Laboratory for Advanced Analytical Technologies, Empa, Swiss Federal Laboratories for Materials Science and Technology, Dübendorf, Switzerland; ^3^ Institute of Environmental Engineering, ETH Zürich, Zürich, Switzerland; ^4^ Institute of Systems Medicine, Chinese Academy of Medical Sciences and Peking Union Medical College, Beijing, China; ^5^ Suzhou Institute of Systems Medicine, Suzhou, China

**Keywords:** AS1411, aptamer, nucleolin, docking, binding mode

## Abstract

AS1411 aptamer can function as a recognition probe to detect the cell surface nucleolin overexpressed in cancer cells, however, little is known about their binding process. This study proposed a feasible binding mode for the first time and provided atomic-level descriptions for the high affinity and specific binding of AS1411. The binding pose predicted by docking was screened using knowledge-based criteria, and a microsecond molecular dynamics (MD) simulation showed the stable existence of the predicted structure in the solution. Structural analysis shows that the unique capping of the 5′ end of AS1411 provides the specific binding with RBD1, and the interactions of hydrogen bond, salt bridge, and water-mediated network between AS1411 and RBD1,2 stabilize the binding. The calculation of per-residue decomposition emphasizes the dominant contribution of *van der Waals* energy and critical residues are screened. Our study provides the molecular basis of this specific binding and can guide rational AS1411-based aptamers design. Further insights require tight collaborations between the experiments and *in silico* studies.

## Introduction

Aptamers are oligonucleotide sequences with a length of around 20–80 bases, including short single-strand DNA (ssDNA) or RNA molecules, first introduced in the 1990s (Debra L. Robertson, 1990; Doug Irvine, 1991), that bind to their specific targets with high affinity and specificity thanks to their stable three-dimensional folding. Due to their lower cost, smaller size (about 10-fold smaller), and easier modification compared to antibodies, aptamers have become ideal recognition candidates for diagnostic and therapeutic agents, targeted drug delivery systems (Jéssica Lopes-Nunes et al., 2020), biosensing probe (Giulia Vindigni et al., 2021; Fuze Jiang et al., 2022; Tong Mo et al., 2022), etc. Aptamers are usually developed *via* systematic evolution of ligands by exponential enrichment (SELEX), while AS1411 is the first non-SELEX anticancer aptamer discovered serendipitously by Paula J. Bates et al. (2009) tested in more than 80 human cell lines and displayed impressive antiproliferative activity by targeted transcription factor BCL2 in most cancer cell types and has entered phase II clinical trials. Thus, aptamers represent a promising strategy in tumor therapy. However, the structural basis of aptamer AS1411 binding with nucleolin is not well understood, which prevents aptamer optimal design or chemical modification for effective applications in biological field.

AS1411 is an antiproliferative G-rich ssDNA, formerly known as AGRO100 and later renamed AS1411 by Antisoma in 2005 and is now also known as ACT-GRO-777 (Bates et al., 2017). Like other G-quadruplexes (GQs), AS1411 is highly polymorphic. Even the intramolecular AS1411 may exhibit distinct topologies. NMR spectroscopy and chromatography have demonstrated that at least eight different monomeric quadruplex structures of AS1411 coexist in K^+^ buffer and can interconvert very slowly at room temperature (Magdalena M. Dailey et al., 2010). And these species have almost identical physical properties but different kinetic stability (Wan Jun Chung et al., 2015). This high complexity makes it challenging to determine its spatial configuration. Encouragingly, several derivatives of AS411 were resolved in recent years. This 26-mer sequence of AS1411, consisting only of guanine and thymine was the optimal, even subtle mutation may lead to the transition of conformations (Paula J. Bates et al., 2009). However, by adding complementary sequences to both ends of AS1411 the conformational polymorphism can be decreased and the core GQ structure be retained. For instance, the extended sequences AS1411-N5 and AS1411-N6 both adopted a single GQ conformation and displayed enhanced affinity, and the added portion seems to have a locking effect (Lee et al., 2010; Fan et al., 2016). Alternatively, by performing chemical modification, such as 2′-deoxyinosine or 5-(N-benzylcarboxyamide)-2′-deoxyuridine the binding and targeting affinity of AS1411 will be increased. It is known that this 26-mer ss-DNA folds mostly into parallel GQ conformation in the existence of K^+^ (Bagheri et al., 2015), but the effects of conformation on the function of specific binding with nucleolin are unclear.

Nucleolin (NCL), the primary molecular target of AS1411, is a multifunctional protein discovered in 1973 and initially called C23 (Larry R. Orrick, 1973; Jing et al., 2020). NCL is ubiquitously distributed in the nucleolus, nucleus, and cytoplasm of the cell (Tajrishi et al., 2011), playing a role in controlling RNA metabolism and ribosome biogenesis. NCL is highly expressed both intracellularly and on the surface of cancer cells, and a 6-fold increase in NCL expression level has been reported in breast and colorectal cancers (Emilio Iturriaga-Goyon et al., 2021). Owing to the overexpression of NCL on the cancer cell surface, AS1411 aptamer can be used as a recognition probe to distinguish cancer cells from normal ones by binding to NCL with selective targeting activity. Currently, it is known that NCL consists of three distinct regions: an acidic N-terminal domain, the middle four RNA-recognition motifs (RBDs or RRMs), and a C-terminal “tail” called GAR or RGG domain, rich in glycine, arginine and phenylalanine residues (Sengodagounder Arumugam et al., 2010). Wherein, RBDs and RGG regions are thought to provide specific RNA binding sites (Vasilyev et al., 2015). However, the high-resolution 3D structure of the full-length protein or the four RBDs is not available. Only NMR solution structures of the RBD1,2 domains from human NCL were resolved (Sengodagounder Arumugam et al., 2010). It is difficult to get a complete picture of intermolecular interactions, especially for the molecules with complicated physical properties like nucleolin and GQ. By searching the RCSB PDB database using nucleolin as the keyword (as of 19.7.2022), we can only retrieve 13 structures determined by NMR spectroscopy and most of which are separate GQs or independent RBD domains contributed by Juli Feigon’s group. Only two structures of hamster nucleolin RBD1,2 complexed with RNA stem-loop (Bouvet et al., 2001; Johansson et al., 2004) were deposited.

Notably, some binding modes of GQ-small molecules have been reported recently. Ortiz de Luzuriaga et al. (2021) pointed out that the different binding between GQs and ligands can be summarized in three modes: end stacking, groove binding, and loop binding mode. Only a few studies have focused on the binding mechanism of GQ and NCL, where the importance of the binding determinants of NCL/GQ and the loop length of GQs has been highlighted but remains controversial. For instance, by investigating a different set of GQ oligonucleotides, Daekyu Sun and co-workers claimed NCL preferentially binds to quadruplexes with shorter loops (Gonzalez et al., 2009). Conversely, two other studies stated that NCL prefers the long loop (Dickerhoff et al., 2019; Saha et al., 2020) and the unique 5′- capping ATG motif of GQ can provide distinctive recognition sites for proteins and small molecules. Likewise, Sara Lago et al. (2017)’s experiments indicated the binding of NCL to GQ directly correlates with the number of G-tracts and heavily relies on GQ loop length. In addition, the solution structure of NCL RBD1,2 binding with b2NRE indicates that the protein specifically recognizes stem-loop pre-rRNA by hydrogen bonds and the stacking interactions mainly involves the *β*-Sheet surface of RBD1,2 and the linker residues (Johansson et al., 2004).

According to these previous studies, some conclusions can be summarized as follows: 1) In the presence of K+, a single major parallel GQ conformation can form and the binding of NCL can enhance the structural stability of GQ. 2) RBD1 and RBD2 do not interact with each other in the free protein, but can interact with the GQ *via* their beta-sheet. 3) The central loop of GQ was a critical contact region, and the length of the loop can affect the binding preference of NCL. 4) The 5-capping motifs of GQ may provide recognition sites for proteins. Although these studies have demonstrated promising results, it is difficult to understand the complex interactions using only experimental approaches. MD simulation-based methods can give the dynamic evolution of the system over time and have been successfully used in many studies. To shed light on the binding mode and the underlying mechanisms, we combined molecular docking and MD simulation to carry out an *in silico* study as a complementary approach to elucidate sufficient details and provide atomic-level descriptions.

## Materials and methods

### Structures preparation

Due to the high complexity mentioned above, no definitive crystal or X-ray diffraction structure of AS1411 was available until 2015 several analogs of AS1411 were solved. Among which, Z-G4 with d[T(GGT)_4_TG(TGG)_3_TGTT] (PDB ID:4U5M) was reported to share the common conformation with AS1411 (Wan Jun Chung et al., 2015), which was taken as a template for modification, resulting in the initial structure of AS1411. Specifically, the penultimate base thymine (T) was mutated to guanine (G) and the thymine bases at both ends were deleted using Discovery Studio Client v19.1.0, thus the preliminary structure of AS1411 was obtained. Then a 20 ns MD simulation was performed at 0.15 mM/L physiological salt concentration (Supplementary Figure S7), and the snapshot closest to the average structure was taken for subsequent docking.

Simultaneously, the coordinates of human NCL RBD1,2 (PDB ID:2KRR) were retrieved from the RCSB Protein Data Bank (RCSB PDB, https://www.rcsb.org/), which included 20 refined structures and some conformations were quite different. Accordingly, choosing the appropriate protein conformation is particularly important for subsequent docking and MD. By comparing different RBD1,2 conformations among the 20 refined structures, two models were selected eventually based on chemical reasoning and the above findings. Specifically, according to the full NMR structure validation report, the 20 refined structures were grouped into four clusters and two single-model clusters (Supplementary Table S3). Based on the results of cluster and according to the separation of RBD1,2 and the structural characteristics of the protein, we selected the representative structures, namely models 1, 4, and 9. Then, by performing a short time simulation, model 1 was exclude because it failed to remain stable within 200 ns MD (Supplementary Figure S6). Finally, model 4 and 9 were input to the docking server together with the previously prepared structure of AS1411 as receptor and ligand, respectively.

### Molecular docking

Most algorithms applied in current docking programs such as AutoDock Vina, Glide, DPDock, and RxDock are not trained for GQ-protein interactions (Dickerhoff et al., 2021). This is because DNA, and especially the non-canonical GQ has unique structural and chemical properties, and their binding sites differ significantly from protein targets. In this work, the prediction was implemented using the HDOCK server (http://huanglab.phys.hust.edu.cn/software/hdocklite/) (Yumeng Yan et al., 2020), which applies an FFT-based scoring function and hierarchical docking algorithms that can support protein-RNA/DNA docking. Since the 3D structures of AS1411 and NCL RBD1,2 are currently available, the risk of inaccurate prediction for GQ can be greatly reduced by directly using the structure files as input. For each pair of receptor and ligand, the rigid-body docking was performed and the server provided the top 10 predicted docking results for visualization. By comparing the docking results (Supplementary Tables S2, S4) and taking into account the potential interaction regions, the third ranked complex of model 9 was identified as the starting point for follow-up work due to the fact that in this binding mode, the 5′- capping of AS1411 stacks with RBD1 and the central loop of AS1411 is close to the *β*-Sheet of RBD2. These interaction regions are consistent with the conclusion mentioned in the introduction section.

### Molecular dynamics simulation

For the complex topology selected from the docking, a MD simulation was performed with the Amber16 (Case et al., 2017) software package, importing the OL15 force field (Rodrigo Galindo-Murillo et al., 2016) for AS1411 and the latest ff19SB (Chuan Tian and Simmerling, 2020) parameters for protein pairing with the OPC water model (Saeed Izadi, 2014). The parameters for ions were from the work of Merz et al. (Sengupta et al., 2021). The complex was embedded in a 71 Å^3^ × 70 Å^3^ × 80 Å^3^ cube box containing OPC water molecules with a minimum distance of 12 Å of solute from the box border. Potassium or chloride ions were added to neutralize the system to a concentration of 150 mM, a concentration that has been verified for the folding of AS1411 (Miranda et al., 2021). Three K^+^ originally located in the cavity between the stacked G-tetrads were not specially treated. Ultimately, the solvated system contained 52,189 atoms.

The system was first minimized for 1,000 steps (500 steps of steepest descent followed by 500 steps of conjugate gradient) with 2 kcal/mol/Å^2^ position restraints on the backbone atoms. Then the restraints were released and another 2,500 steps (1,000 steps of steepest descent followed by 1,500 steps of conjugate gradient) were performed to further equilibrate the system. Afterward, the system was heated gradually from 0 to 300 K over 500 ps with restrains on backbone atoms under the control of Langevin thermostat (Richard J. Loncharich, 1992) and then equilibrated for 5 ns in the NVT ensemble without restrains. Finally, a 1 μs production run was carried out under the NPT ensemble. During the production phase, Berendsen barostat (H. J. C. Berendsen et al., 1984) was used to control the pressure at 1 atm. A 10 Å cut-off was set for nonbonded interactions, and the Particle Mesh Ewald method (PME) (T. E. Cheatham et al., 1995) was used to calculate the electrostatic interactions. SHAKE (Kollman, 1992) constraints were applied to all covalent bonds involving hydrogens. Trajectory analysis was performed using the cpptraj (Roe and Cheatham, 2013) module of AMBER16 and the MM/GBSA method was applied to calculate the solvated free energy and pairwise interaction energy. The salt bridge analysis was implemented with the online tool PLIP (Adasme et al., 2021) and VMD 1.9.4 program (William Humphrey, 1996) was used for visualization.

## Results

### Structural features and system equilibrium

**FIGURE 1:**
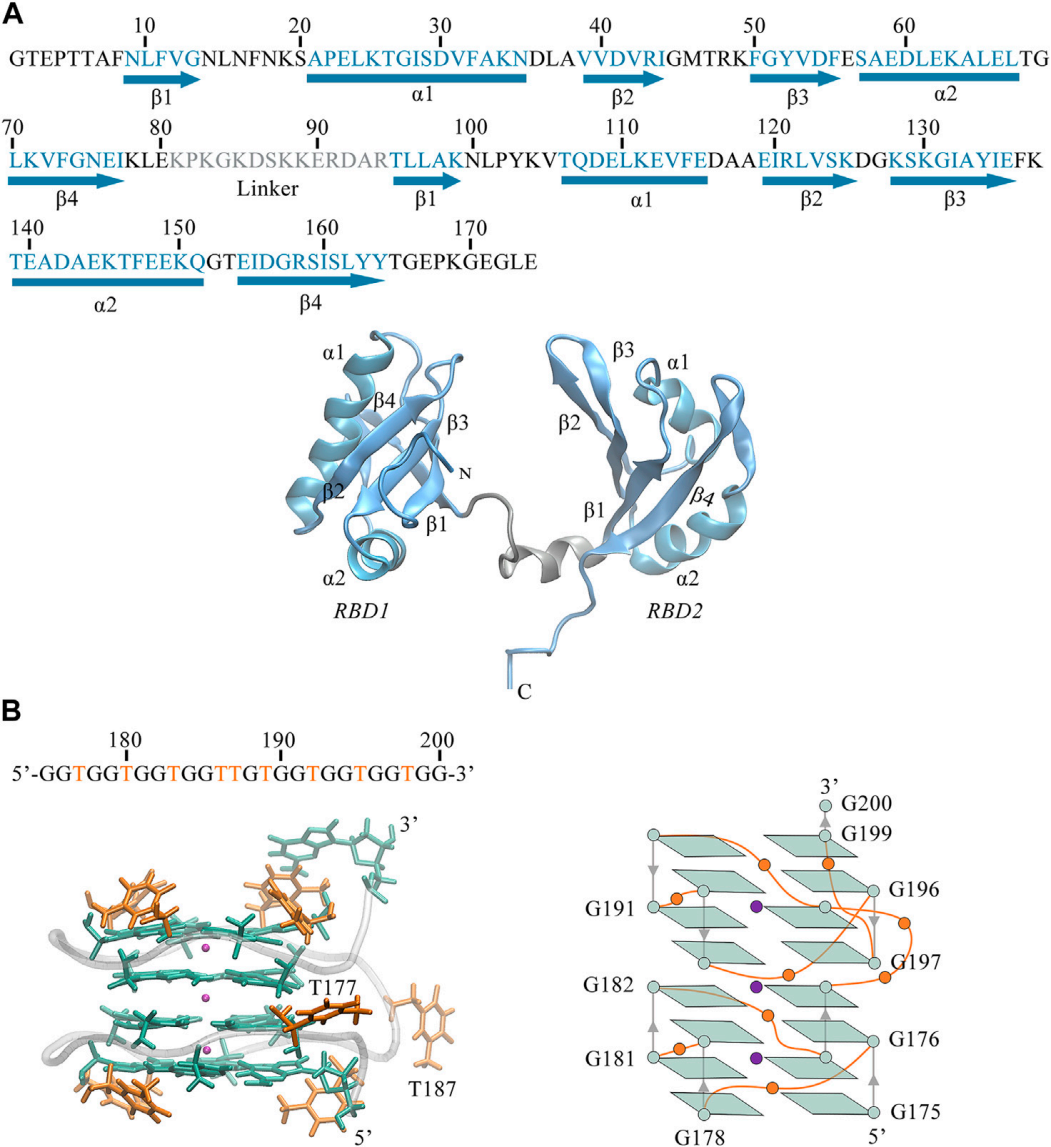
**(A)** Sequence and 3D structure of human NCL RBD1,2 (PDB ID:2KRR, model-9). The secondary structure sequence of α -Helix and β -Sheet are in light blue and indicated below the sequence. The gray region (81–94) corresponds to the linker connecting RBD1 (1–80) and RBD2 (95–174), where residues 88–93 of linker are in helix conformation. Both RBDs adopt β1α1β2β3α2β4 fold and are displayed in NewCartoon mode. **(B)** Sequence and 3D structures of AS1411 extracted from the 20-ns trajectory. For clarity, the protein and DNA are numbered consecutively, accordingly, the 26nt AS1411 is numbered 175 to 200 with thymine in the loops are colored in orange. The lower left panel is the representation of AS1411, wherein, the guanine bases are colored in green, the thymine bases in orange, and the backbone atoms in bases are not shown. The phosphate backbone is shown in silver Tube mode and three K^+^ ions between the tetrads are shown in violet VDW mode. The flipping bases T177 and T187 are highlighted with labels. The corresponding simplified schematic is shown in the lower right. Except for the terminal G200, the 16 guanines form the following 4-layer tetrads: G175, G178, G181, G184; G176, G179, G182, G185; G188, G191, G194, G197; and G190, G193, G196, G199; The arrows represent the progression of 5′ to 3′ strand.

As shown in Figure 1, we can see the unique features of our target system. The 26-mer sequence d(GGTGGTGGTGGTTGTGGTGGTGGTGG) of AS1411 forms a stable parallel left-hand GQ (see Figure 1B), characterized by the compact stacking of four-layer G-tetrads (also called G-Quartets), in which four guanines are linked through Hoogsteen-type hydrogen bonds (H-bond). The O6 atoms of guanines are oriented to the center of the structure, creating an electronegative channel, which is stabilized by the coordination of central K^+^. Such G-tetrads stack on one another to serve as two building blocks, within each block, strands are connected by single-nucleotide thymine loops except for the continuous T186 and T187 in the central loop. The average structure was obtained over the last 20 ns trajectory, and the snapshot with the smallest RMSD value compared to the average structure was selected as the representative structure. The representative structure was superimposed with the X-ray structure of Z-G4, showing a high degree of concordance (Supplementary Figure S1A), suggesting that the OL15 force field provides satisfactory modeling for AS1411.

Also, we can observe the primary structure of RBD1,2 consists of 174 residues (see Figure 1A). Both RBDs adopt the expected β1α1β2β3α2β4 fold and are connected by a 14-residue linkage region, thus forming a binding pocket in the middle of the sandwich structure. By comparison, the two domains have similar conformations but are not symmetrical. In the RBD1 domain, the α1-Helix is longer and the *β*-Sheets are shorter, resulting in longer disordered linkages region between them. Notably, these random loops in RBD1 are located primarily at the entrance of the pocket, while in the RBD2 on the other side, this region is occupied by the long and ordered β2, β3. Therefore, it is reasonable to speculate different interactions due to these differences in the spatial structures of RBD1 and RBD2, which will be further discussed in the following sections.

**FIGURE 2:**
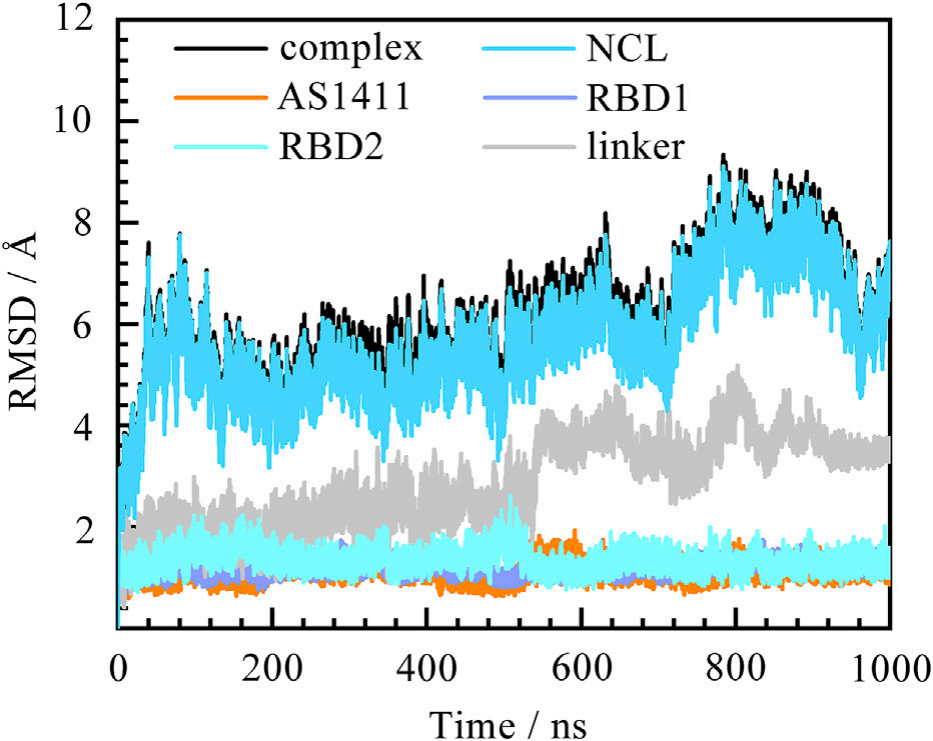
Root mean square deviation (RMSD) of the system over 1,000 ns MD simulation. The complex, protein NCL RBD1,2, AS1411, and individual RBD1, RDB2, and linker are represented by black, blue, orange, purple, cyan, and grey, respectively. The eight residues at the ends of the protein were not included in the calculations to avoid the “end effect” bias.

Before figuring out how these features contribute to the binding, the thermodynamic equilibrium for the predicted binding pose was first evaluated. The root mean square deviations (RMSD) of heavy atoms were calculated with reference to the initial conformation. As depicted in Figure 2, the RMSDs of the system increase quickly within the first 50 ns and then remain stable for the rest of the simulation. The average fluctuations of the complex, NCL RBD1,2 and AS1411 are 5.93 ± 1.18, 5.67 ± 1.17, and 1.12 ± 0.15 Å, respectively. The small fluctuations magnitude within 1.2 Å indicates the system has reached equilibrium. The overall trend of the system is consistent with that of protein, while the individual GQ, RBD1, and RBD2 regions show little conformational change, only the linker region connecting RBD1 and RBD2 showing some fluctuations. Further investigation suggests that these variations can be attributed to the relative motion of the two domains by aligning the structures extracted before and after the curve transition in the plot (Supplementary Figures S2, S5).

In addition, the root mean square fluctuations of each residue (RMSF) were also calculated. The results confirmed that the main fluctuations are concentrated in the disordered loop regions linking the β -Sheets, with the amplitude varying between ∼2 and 6 Å (Supplementary Figure S3) and the β2 and β3 of RBD2 are quite stable. On the other hand, except for the most fluctuating T77 (4 Å), the RMSF of the other bases is smaller, fluctuating around 2 Å, and all peaks correspond to the thymine loops of GQ, implying that the guanines to form the G-tetrads are rather stable and the binding of protein stabilizes the structure of GQ.

### Binding mode analysis

**FIGURE 3:**
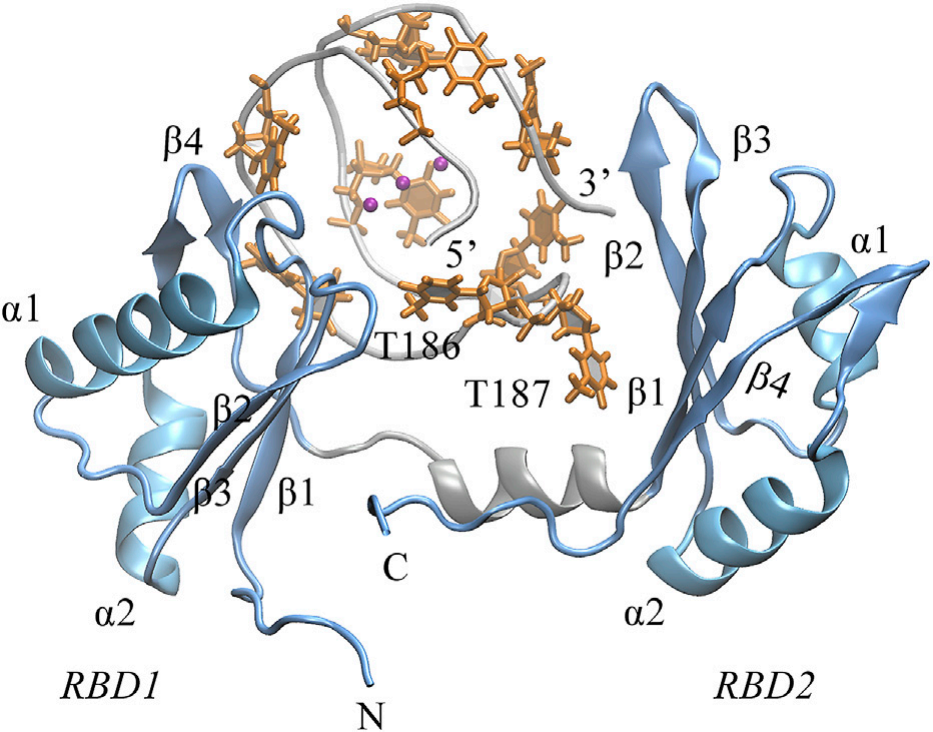
The binding pose of the complex was extracted from the last 200 ns equilibrium trajectory. The topology of protein is indicated in light blue NewCartoon mode with the secondary structure labeled. The phosphate backbone of AS1411 is shown in silver Tube mode. The external capping thymine bases are colored in orange, and the positions of the central loops T186-T187 are annotated in the plot.

**FIGURE 4:**
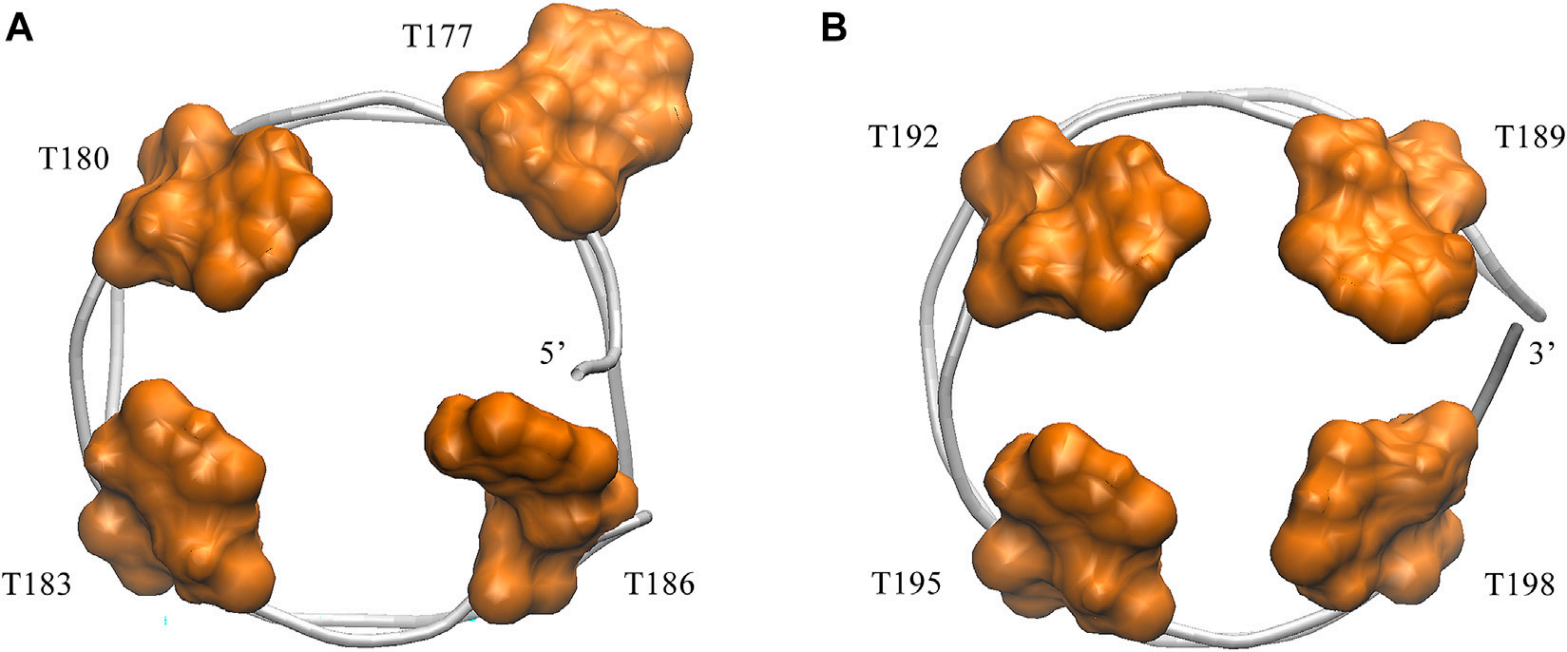
Capping of AS1411in the complex. **(A)** Bottom view of the 5′ capping. **(B)** Top view of the 3′ capping. Orange thymine bases are labeled with their numbers and shown in surface mode. Phosphate backbone is colored in silver.

Next, we turned to the binding mode of AS1411 with RBD1,2 as shown in Figure 3. The association conformation exhibits certain significant features. First of all, the RBD1 of NCL was stacked with the 5′ end of AS1411. From the structural point of view, AS1411 consists of 17 guanines and nine thymines (see Figure 1B), among which, guanines primarily form the four-layer tetrads, while the other eight thymines collapse onto the terminal G-tetrads to form the external capping at the two ends (Figure 4). Specifically, T189, T192, T195, and T198 at 3′ end form a complete capping, while at the 5′ end, thymine T177 does not get involved in the capping of T180, T183, and T186. It flips out and has more degrees of freedom due to no contact with the protein, which also explains its significant fluctuation in RMSF. This unique capping-deficient mode and the accessible grooves in AS1411 (Figures 3, 4) can provide specific contact sites with NCL. Meanwhile, the 3′ end and central loop of GQ inserted into the pocket may form contacts with RBD2 to further stabilize the binding. Once these regions of AS1411 form stable contacts with RBD1 and RBD2, this delicate scaffold can provide anchors for specific recognition, resulting in stable binding.

**FIGURE 5:**
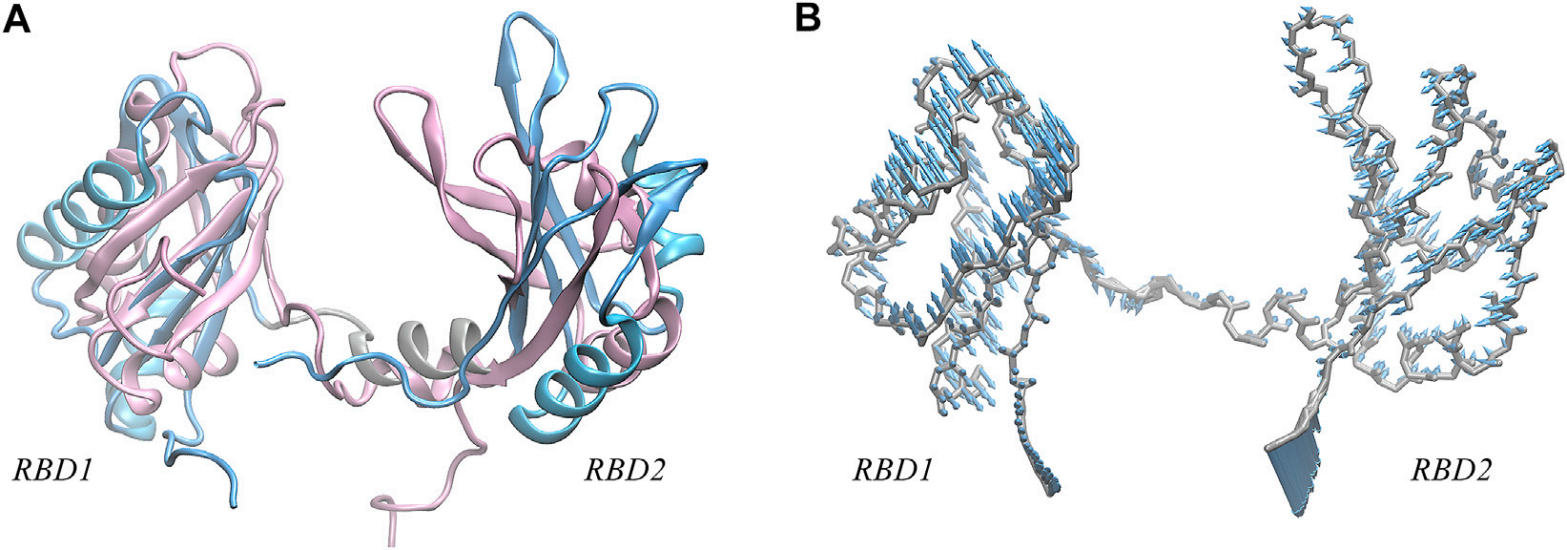
**(A)** Superposition of the NMR structure of model 9 (pink) and the structure (light blue) of NCL extracted from the equilibrium complex, shown in New Cartoon mode. **(B)** Porcupine plots of the first principal component, describing the motion pattern of 59% of the protein. PCA analysis of the protein was performed using the cpptraj module in AmberTools based on 25,000 frames from the 1,000 ns trajectory.

Further, we investigate the conformational change of the system upon binding. By examining the conformation of AS1411 in the complex, a good agreement with the initial structure was shown (Supplementary Figure S1B), indicating that AS1411 did not undergo conformational adjustment upon binding. Also, the local conformation of the protein at the binding interface was investigated. According to the superimposition of protein conformations before and after binding (see Figure 5A), it can be observed a wider open pocket, implying a local conformational adjustment upon binding. The motion mode obtained by principal components analysis (PCA) analysis (Galindo-Murillo et al., 2014) confirmed the relative motion between the domains of the protein (see Figure 5B), leading to the opening of the pocket and a localized conformational adjustment of the two domains. However, since our simulations are based on rigid docking, whether this binding is the result of induced-fit or conformational change mechanism (Cera, 2012) requires further investigation. To better understand the structural requirements driving the binding and to characterize the thermodynamics and kinetics, the interactions at the binding interface were further analyzed.

### Interactions in the interface

To elucidate the specific recognition, a statistical analysis of the direct interactions at the binding interface was performed. Hydrogen bond (H-bond) and salt bridge interactions were first calculated, applying the geometric criteria with an angle cutoff of 135° and distance cutoff of 3.0 Å for H-bonds and a distance cutoff of 4.0 Å for the salt bridge.

**FIGURE 6:**
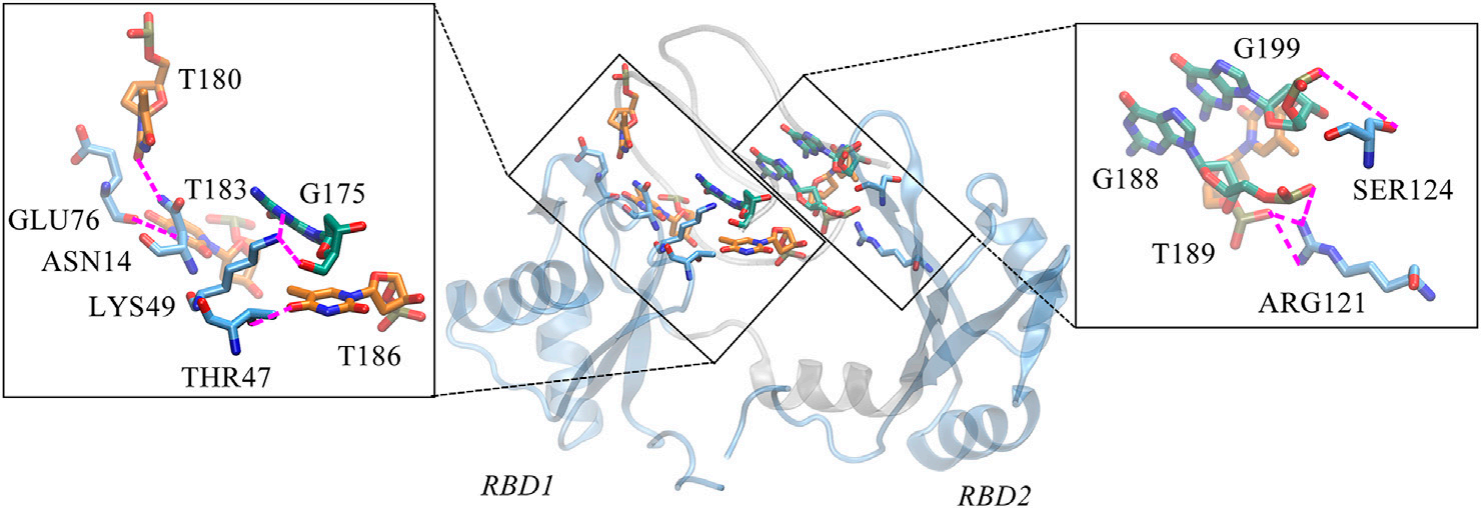
Key hydrogen bond interactions at the interface. Magenta dashed lines indicate hydrogen bonding interactions and the residues involved are shown in Licorice mode. The colors of amino acids, T-bases and G-bases are indicated in blue, orange and green, respectively, and C- atoms are shown in the color corresponding to the backbone, Atoms O, N, and P are shown in red, blue, and tan, respectively. For clarity, the H atoms are not shown. This conformation is extracted from the last 200 ns trajectory.

Unexpectedly, only a small percentage of hydrogen bonds and salt bridges were obtained (Table 1; Supplementary Table S1). Figure 6 presents the major H-bonds. It is noted that the bases at the 5′ end, i.e., T180, T183, and T186, their side chains form stable hydrogen bonds with residues ASN14, GLU76, and THR47 of RBD1, respectively. At the same time, the long side chain of LYS49 inserts and forms a stable bifurcated hydrogen bond (Isabel Rozas, 1998) with the terminal base G175. The high occupancy of H-bonds indicates the specific interactions (Maoxuan Lin, 2019) are quite stable. All these residues are located in the flexible linkage region between the *β*-Sheets of RBD1 and these stable H-bonds restrain residue fluctuations and are the major contributors to the specific binding. On the other hand, at the contact interface with RBD2, a prominent basic amino acid ARG121 can be observed, which is located at β2 of RBD2 with its long side chain sticking out and close to AS1411, leading to multiple H-bonds with the backbone atoms of G188 and T189. In addition, according to the investigation of the salt bridge (Supplementary Table S1; Supplementary Figure S4), ARG121 also contacts T189 by salt bridge interaction with a high probability of 92.2%. At the same time, residues Lys83 and Lys85 in the linker region form transient salt bridges with the phosphate backbone of G184-G185. Thus, taken together, the stable H-bonds at the 5′ end of AS1411 provide specific recognition, and interactions of the backbone H-bonds and salt bridges with the linker and RBD2 regions enhance the binding, and finally, these interactions stabilize the structure of the complex.

**TABLE 1:** Hydrogen bond interactions at the interface. All the data are calculated based on the 5,000 snapshots extracted from the last 200 ns trajectory, using the geometric criteria with an angle cutoff of 135° and distance cutoff of 3.0 Å. The occupancy was truncated with a cutoff of 20%.

Acceptor	Donor	Occupancy (%)	NCL
G175@O5′	LYS49@NZ	68.40	RBD1
G175@N3	LYS49@NZ	63.46	
T180@O2	ASN14@ND2	61.80	
T180@O2	GLU76@N	25.90	
GLU76@O	T183@N3	41.60	
T186@O4	THR47@OG1	81.94	
G188@OP1	ARG121@NH2	75.30	RBD2
T189@OP2	ARG121@NH2	87.42	
T189@OP2	ARG121@NH1	50.22	
G199@OP2	SER124@OG	36.30	
G200@OP1	SER124@OG	21.44	

**FIGURE 7:**
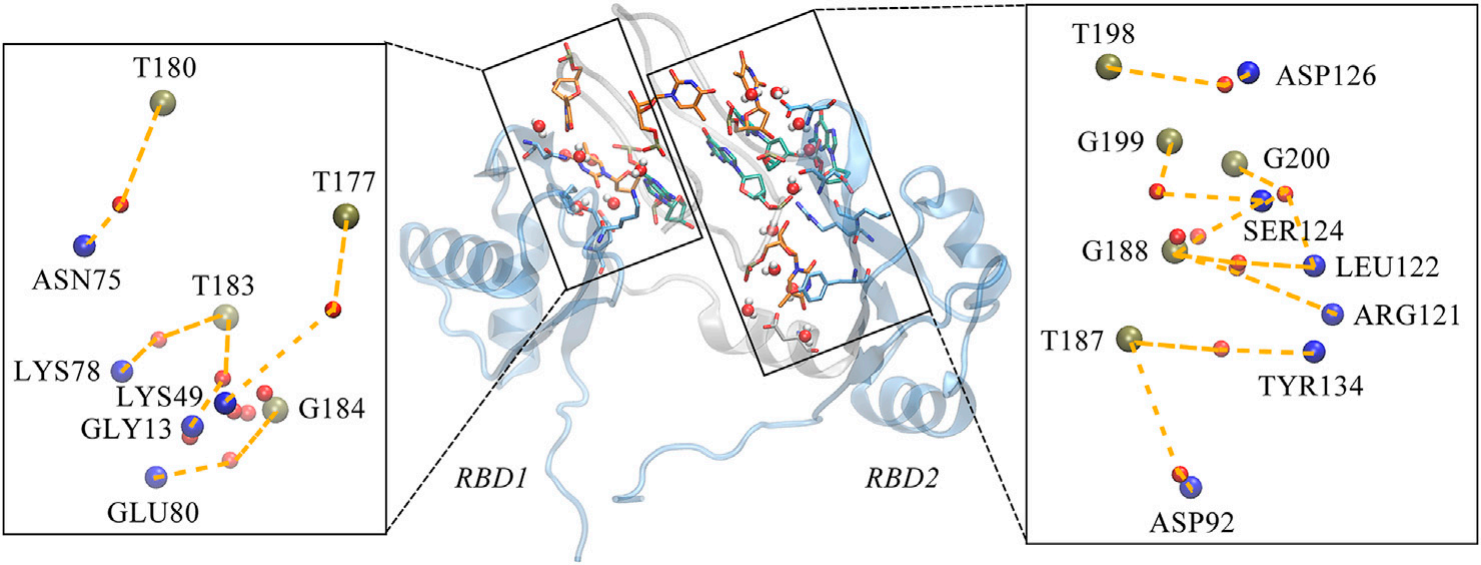
Water-mediated hydrogen bond interactions at the interface. The binding conformation in the middle of figure depicts the major contact region, the interacting bases and residues are shown in the same way as the hydrogen bond diagram, with the water molecules are shown in *VDW* mode, where the oxygen atom is shown in red and the two hydrogen atoms in white. Details of the interactions are given in black rectangular boxes on either side of the conformation diagram and the interactions are indicated by the orange dashed lines. For clarity, the bases in AS1411, the residues in the protein and the water molecules are shown as simplified tan, blue and red balls, respectively. This conformation is extracted from the last 200 ns trajectory.

Considering the extensive existence of water molecules in most of the reported GQ structures, and potential critical roles at the interface, further investigation was carried out for water-mediated interactions. The results show an extensive network of interactions mediated by water molecules. As shown in Table 2 and Figure 7. The water-mediated interactions at the 5′ end block involve T177, T180, T183, and G184, and likewise, the protein residues that interact with T180, G184, and T183 are primarily in the disordered loops of RBD1. In contrast, more water molecules are sandwiched between the 3′ end block and RBD2, mediating a local hydrogen bond network. Specifically, residue T187, flipping out of the planar forms two mediated interactions with TYR134 and ASP92, which contribute the most according to the occupancy. Two water molecules adjacent to residue G188 bridge the side chain by hydrogen bonds with ARG121 and LEU122. In addition, the T198-G199-G200 triad at the 3′ end all forms extensive water-mediated hydrogen bonds with residues in β2 and β3 of RBD2. From this point of view, it can be inferred that the issue of “end fraying” (Islam et al., 2020) for AS1411 in this work is not significant, almost every base plays a role in the current binding mode.

**TABLE 2:** Water-mediated hydrogen bonds of protein-GQ. All the data are calculated based on the 5,000 snapshots extracted from the last 200 ns trajectory, using a cutoff of 20% for occupancy.

AS1411	NCL	Occupancy (%)	
T177	LYS49	23.38	RBD1
T180	ASN75	33.84	
T183	GLY13	33.00	
T183	GLY13, LYS78	40.94	
G184	GLU80	60.42	
T187	ASP92	151.52	Linker
T187	TYR134	30.62	RBD2
G188	ARG121, LEU122	24.16	
G188	LEU122	23.82	
T198	ASP126	73.36	
G199	SER124	38.98	
G200	LEU122	66.30	
G188, G200	SER124	21.56	

Collectively, although these non-covalent interactions are relatively weak, the small interactions including hydrogen bonds, salt bridges, and especially water-mediated interactions may add up to make an important contribution to the specificity and high affinity.

### Free energy calculation and per residue decomposition

Considering the flexibility of protein structure, we only focus on the assessment of binding free energy (Wang et al., 2019) which favors binding and the entropy-enthalpy compensation is not discussed here for the present, which is computationally expensive and may tend to have a large margin of error that introduces significant uncertainty in the result. In this work, the MM/GBSA model implemented in the AMBER16 software package was used to obtain binding free energy.

As shown in Table 3, the result of binding free energy is −109.30 ± 8.14 kcal/mol, this large negative value indicates that interactions between the two molecules make them tightly bound. The sum of ΔE_*vdW*_ and ΔG_SUR_ is −118.82 kcal/mol, while the sum of ΔE_ELE_ and ΔG_GB_ is 9.52 kcal/mol, which means the binding derives primarily from the favorable non-polar *van der Waals* (*vdW*) attraction. Although the electrostatic interactions between the solute molecules are strong (−135.02 kcal/mol), the electrostatic interactions between AS1411 and the solvent are even stronger. Due to the cancellation of positive and negative charge effects, the electrostatic effects, on the whole, are unfavorable to the binding. The driving force of the association is dominated by nonpolar interactions.

**TABLE 3:** Binding free energy of AS1411-NCL. All the data are calculated based on the 500 snapshots extracted from the last 200 ns trajectory. The unit is kcal/mol.

∆G_*vdw*_	∆G_SUR_	∆G_ele_	∆G_GB_	∆G_non-pol_	∆G_pol_	∆G_bind_
−105.67 ± 7.38	−13.15 ± 0.84	−135.02 ± 39.47	144.54 ± 37.45	−118.82	9.52	−109.30 ± 8.14

**FIGURE 8:**
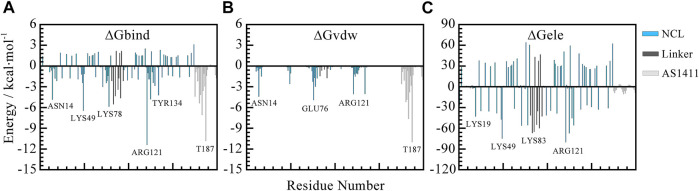
Per-residue decomposition contributions to free energy. **(A)** Total free energy contributions. **(B)** *van der Waals* energy contributions. **(C)** Electrostatic energy contributions. The blue, gray, and silver bars represent RBD1 and RBD2, the linker region, and AS1411, respectively. All the data are calculated based on the 500 snapshots extracted from the last 200 ns trajectory.

To further screen the key residues, per-residue contributions were decomposed. Figure 8 depicted the per-residue energy components contributing to binding free energies. From this, we can see the consistent trend of total free energy and *vdW* term. Specifically, the residues that contribute most are mainly thymine bases including T180, T183, T187 in AS1411 and residues in the flexible linkage of protein such as ASN14, LYS76, ARG121 etc. which is consistent with the previous analysis.

To sum up, the unique capping at the 5′ end of AS1411 provides specific recognition with RBD1, and the hydrogen bonds and the water-mediate network further stabilize the structure. While the dominant binding of AS1411 with RBD1,2 was driven by the nonpolar *van der Waals* absorption.

## Discussion

AS1411 aptamer has been widely used for its ability to distinguish cancer cells from normal ones by binding to cell surface NCL (Jing et al., 2020). However, the mechanism underlying the NCL-targeting ability of AS1411 is not completely understood. In this work, we reported a potential binding mode for the first time and provided a reasonable interpretation for the high affinity and specific binding at the atomic level. The 1-μs simulation confirmed the stability of the binding conformation. Moreover, a complete picture of intermolecular interactions was given and the key residues were screened out. Specially, the 5′-capping of AS1411 provides specific H-bond interactions with RBD1. Although the RBD1 and RBD2 have similar β1α1β2β3α2β4 fold but they are not symmetrical and form distinct interactions with AS1411, especially the basic amino acid ARG121 located at β2 of RBD2 forms multiple H-bonds with the central loop of AS1411. These observations are in good agreement with the conclusion summarized in introduction section. However, it must be acknowledged some limitations that may affect our *in silico* study, mainly arise from the following aspects:

Firstly, the available experimental data are extremely limited. In this work, the only experimental data that we have is the X-ray structure of the AS1411 derivative and a set of 20 solution NMR structures of human NCL RBD1,2. Wherein, the presence of K^+^ can stabilize the GQ structure of AS1411, however, NCL protein exists in an ensemble of conformations, the NMR solution structures of the human NCL RBD1,2 (2KRR) include 20 refined structures and some conformations are quite different. Very little is known about the specific role of this protein in cancer and it is thought that only certain forms of NCL can be affected by AS1411 (Paula J. Bates et al., 2009).

Secondly, the accuracy of docking is limited by the scoring functions and should be used with caution. Encouragingly, HDOCK servers, used in this work, can support protein-RNA/DNA docking by integrating an intrinsic scoring function. Although the high quality of screened binding pose was proved by a subsequent MD study and obtained satisfactory results, it needs to be validated by future experimental measurements.

Thirdly, the accuracy of the force-field description of GQ structures. As is known that reliable results of MD simulation highly depend on accurate force fields (FF). While in classical force field parameters are not optimized specifically for GQ, and it does not include electronic polarization, which may be a possible limitation in simulations of GQs. According to the systematical evaluations on the five commonly used FFs: parmbsc0, parmbsc1, OL15, and Drude 2017 (Li et al., 2021; Ortiz de Luzuriaga et al., 2021), Drude 2017 may be a good choice in certain protein-GQs complex but may overestimate the hydrogen bonds. While the updated OL15 FF has been widely used in protein-DNA interactions and currently the descriptions of GQs have been improved (Perez et al., 2007; Islam et al., 2017; Giambasu et al., 2019). Our simulation with OL15 FF and OPC water model shows satisfactory results and is in good agreement with the current study.

Taken together, despite all the possible limitations, which can be substantially reduced by our manual screening and careful selection of force field parameters based on the existing research conclusion. The results shed light on the binding mechanism and lay the foundation for the development of more efficient AS1411 aptamers.

## Data Availability

The original contributions presented in the study are included in the article/Supplementary Material, further inquiries can be directed to the corresponding author.
