# Supplementary material for: Insights into the binding mode of AS1411 aptamer to nucleolin
Source: Front Mol Biosci. 2022 Oct 3;9:1025313. doi: 10.3389/fmolb.2022.1025313 (PMC9574071; doi:10.3389/fmolb.2022.1025313)
Supplement: Supplementary file 1 [file DataSheet1.docx]

Supplementary Material


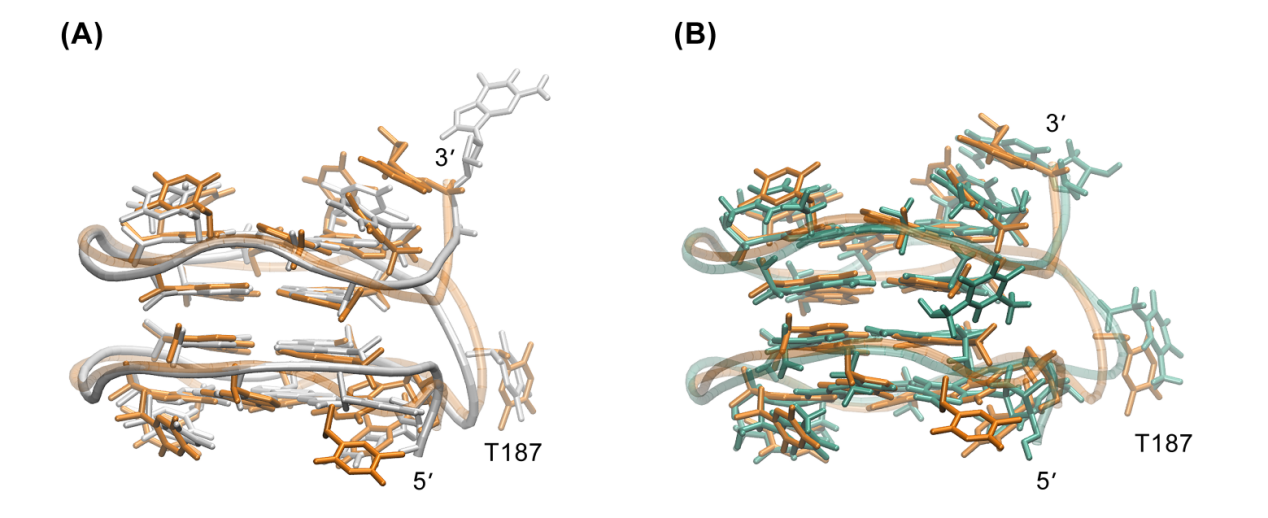


**Supplementary Figure S1.** (A) Superposition diagram of the equilibrium AS1411 structure (orange) and the x-ray structure of Z-G4 (PDB ID:4U5M) (silver). wherein, the equilibrium structure is derived from the manually processed AS1411. For comparison, the two residues thymine at the terminus of 28nt Z-G4 are not shown. (B) Superposition diagram of the AS1411 structure extracted from the complex (green) with the manually processed AS1411 structure (orange).

**
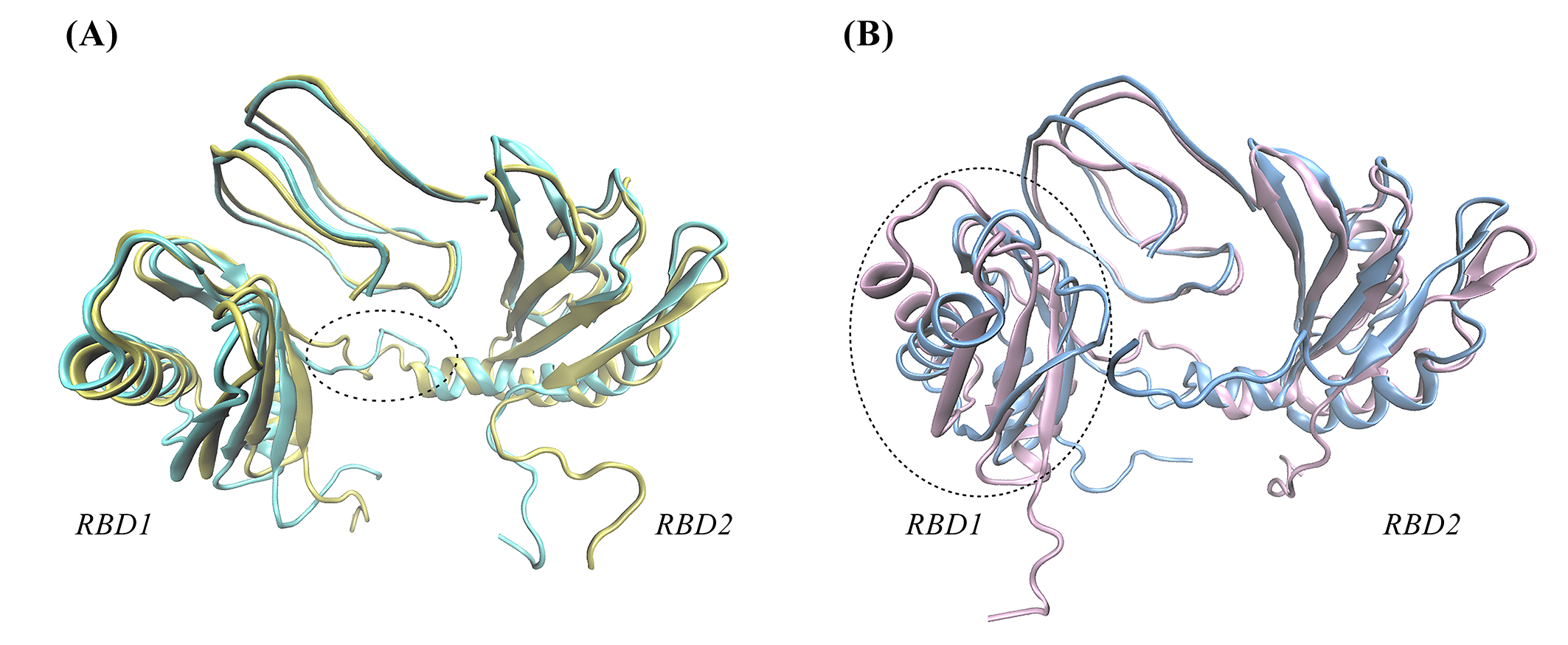
**

**Supplementary Figure S2**. (A) Superposition conformations of the protein corresponding to the fluctuations in RMSD values of the linker region, before and after ~550 ns. The yellow and cyan colors indicate the average structure at 30 ns (510-540 ns) before and 30 ns (550-580 ns) after the jump, respectively. (B) Superposition conformation of the protein corresponding to the fluctuations in RMSD values of NCL RBD1,2, before and after ~700 ns. The pink and blue colors indicate the average structure at 30 ns (650-680 ns) before and 30 ns (730-760 ns) after the jump, respectively.


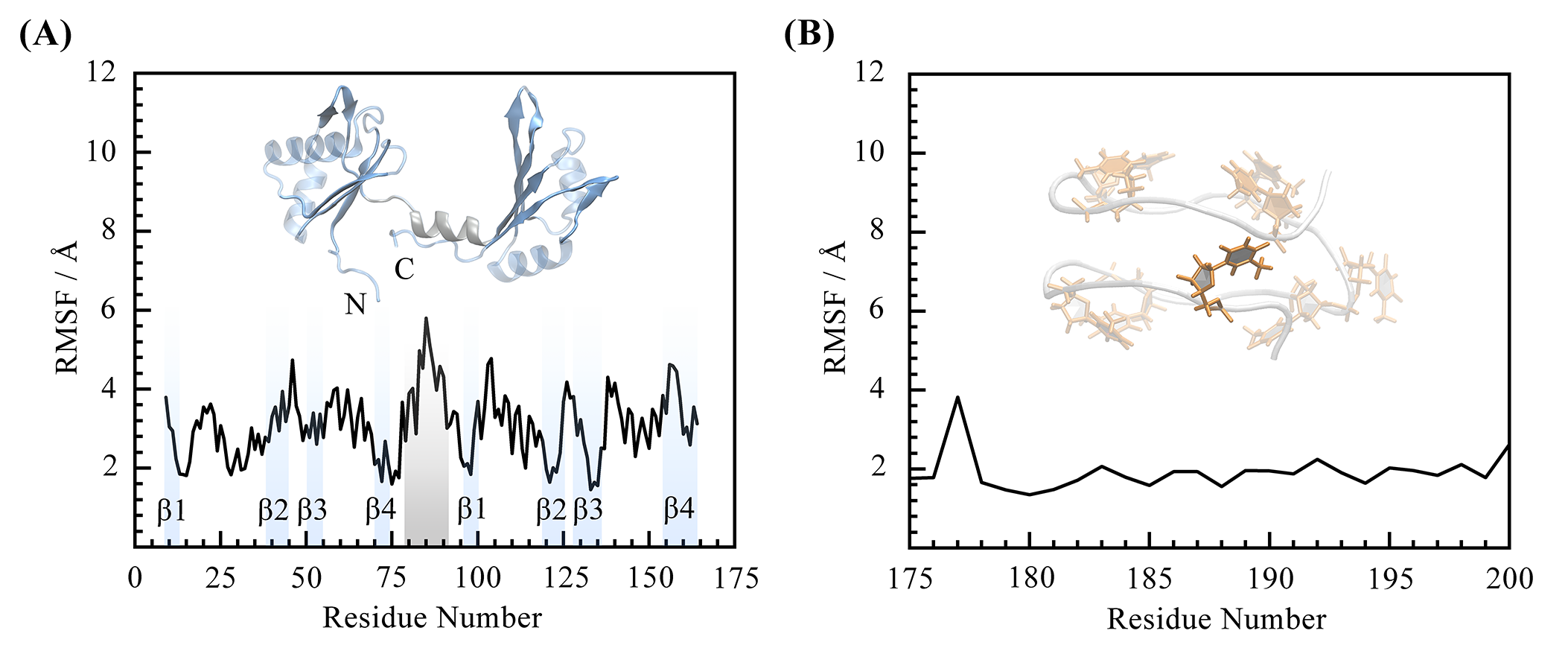


**Supplementary Figure S3.** (A) Per-residue RMSF of RBD1,2. Key secondary structures are highlighted with colored block and labels. (B) Per-nucleotide RMSF of AS1411. The peak value of T177 corresponds to the highlighted base shown in the conformation above the curves.


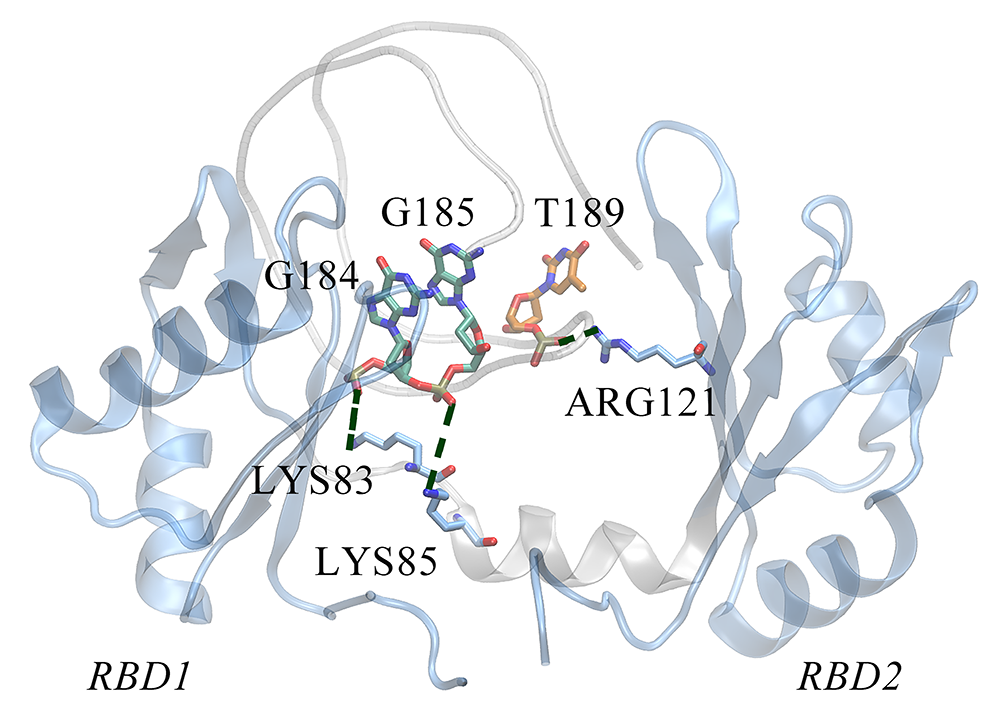


**Supplementary Figure S4.** Key salt bridge interaction at the interface. The salt bridges are connected by green dashed lines. Residues involved are shown in Licorice mode. The colors of amino acids, T-bases, and G-bases are indicated in blue, orange, and green, respectively, and C- atoms are shown in the color corresponding to the backbone, Atoms O, N, and P are shown in red, blue, and tan, respectively. For clarity, the H atoms are not shown. This conformation is extracted from the last 200 ns trajectory.


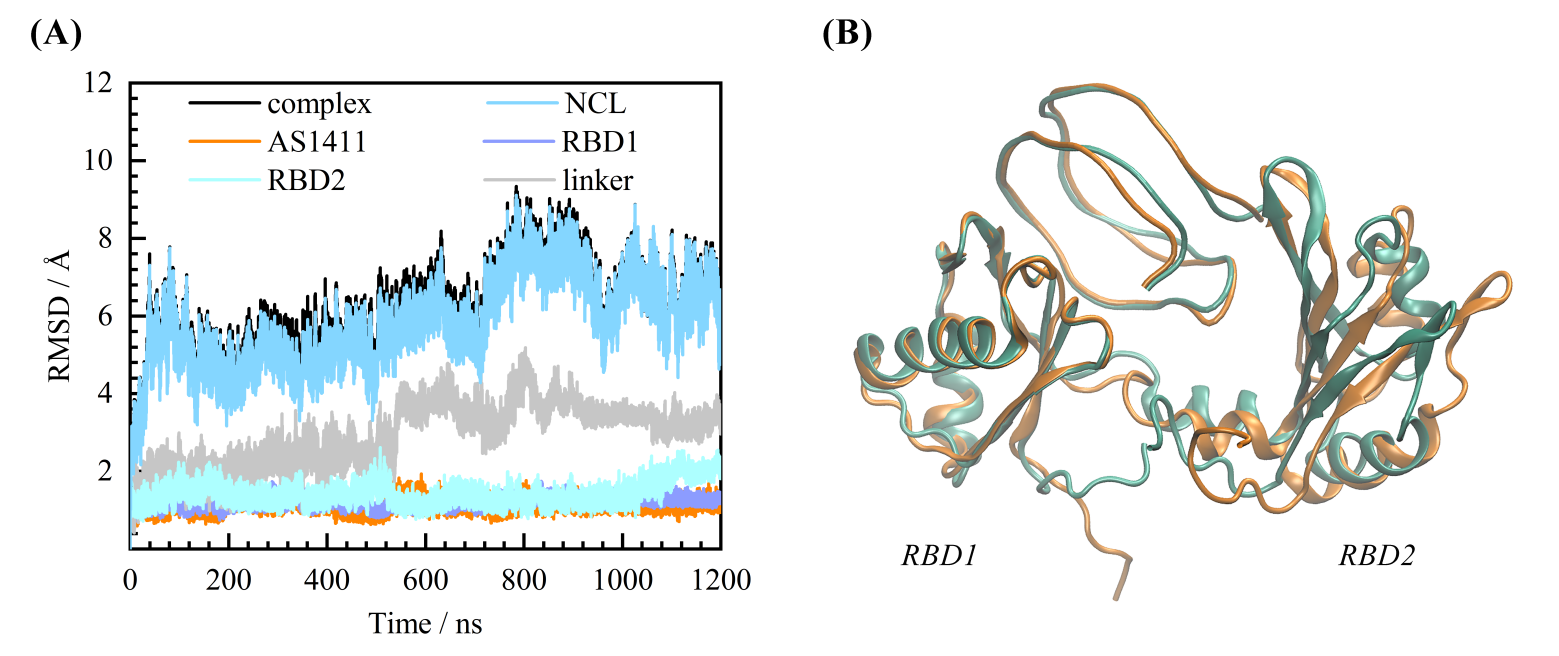


**Supplementary Figure S5.** (A) Root mean square deviation (RMSD) of the system over 1200 ns MD simulation. complex, protein, NCL RBD1,2, AS1411, and individual RBD1, RDB2, and linker are represented by black, blue, orange, purple, cyan, and grey, respectively. The 8 residues at the ends of the protein were not included in the calculations to avoid the “end-effect” bias. (B) Superposition conformations of the complex corresponding to the simulated results before and after ~950 ns. The orange and green colors indicate the average structure at 920-950 ns and 970-1000 ns, respectively.


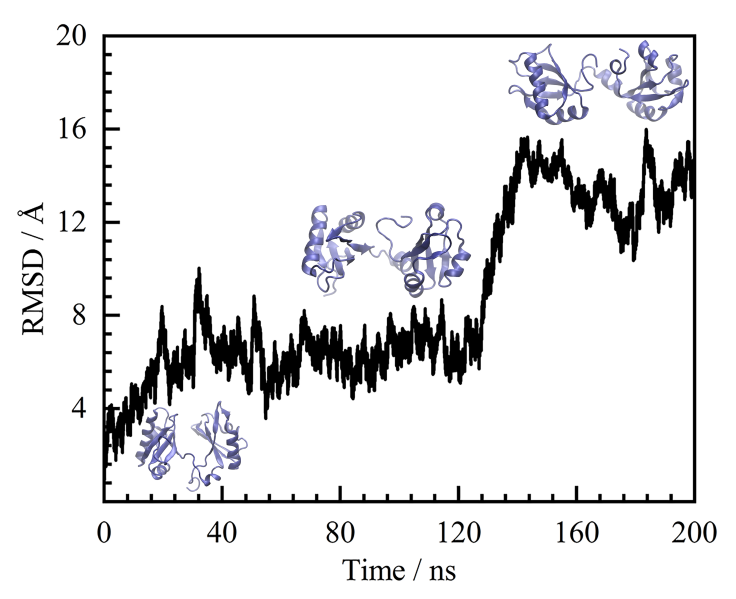


**Supplementary Figure S6.** Root mean square deviation (RMSD) of the free protein of model1 over 200 ns MD simulation. The three subgraphs from left to right correspond to the representative structures of 0 ns (the initial structure), 90 - 120 ns and 140 -170 ns, respectively. The representative structure was selected as the snapshot closest to the average structure.


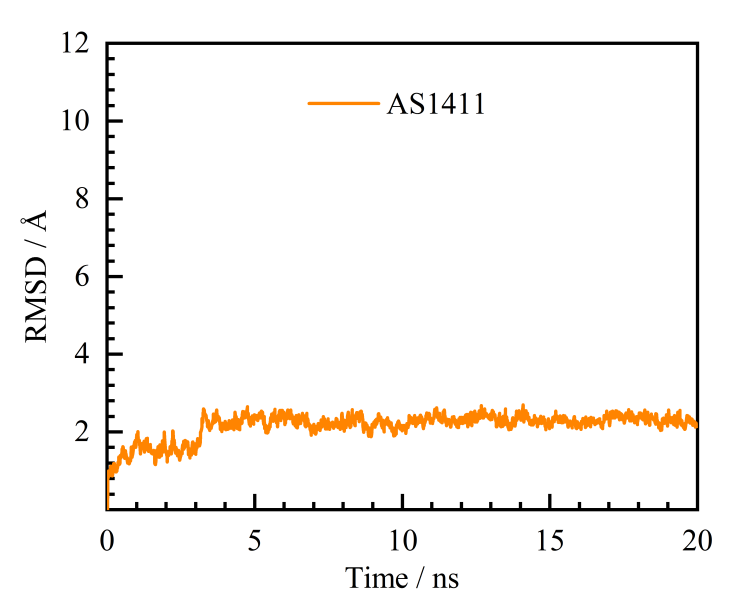


**Supplementary Figure S7.** Root mean square deviation (RMSD) of AS1411 over 20 ns MD simulation. The initial structure was derived from the manually processed AS1411.

**Supplementary Table S1.** Salt bridge interactions at the interface. All the data are calculated based on the 5000 snapshots extracted from the last 200 ns trajectory, using the geometric criteria with a distance cutoff of 4.0 Å. The occupancy was truncated with a cutoff of 20%.

| Acidic | Basic | Occupancy(%) | NCL |
| --- | --- | --- | --- |
| G184 | LYS83 | 48.44 | Linker |
| G185 | LYS85 | 31.46 |  |
| T189 | ARG121 | 92.20 | RBD2 |

**Supplementary Table S2.** The top 10 predicted docking modes using model 9 of protein and AS1411 as input files of HDOCK (Yan, Tao et al. 2020). The proteins are shown in blue cartoon mode and GQs are represented in silver Tube mode. The minimum energy value from docking is shown under each structure with a unit of kcal/mol.

|  | structure |  | structure |
| --- | --- | --- | --- |
| 1 | 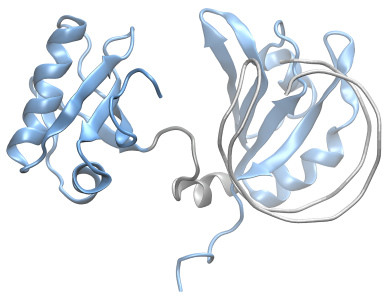  -245.70 | 2 | 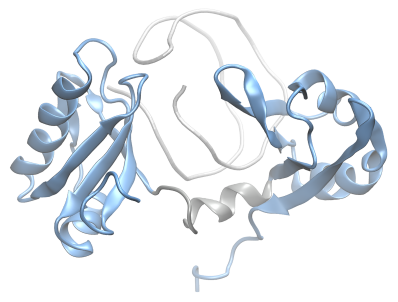  -244.37 |
| 3 | 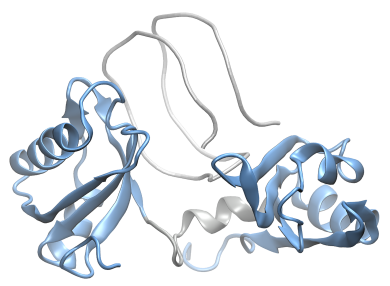  -239.74 | 4 | 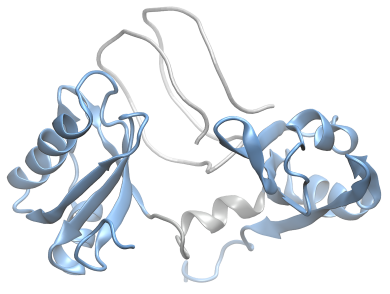  -237.16 |
| 5 | 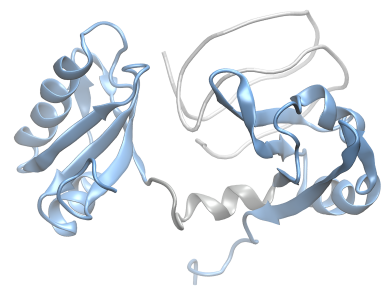  -225.76 | 6 | 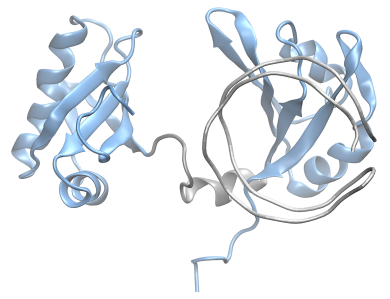  -220.86 |
| 7 | 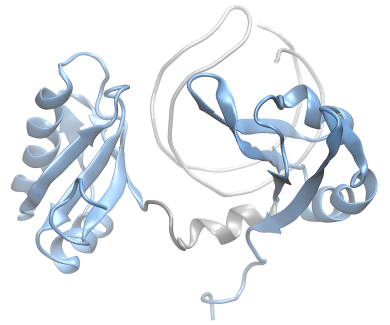  -218.93 | 8 | 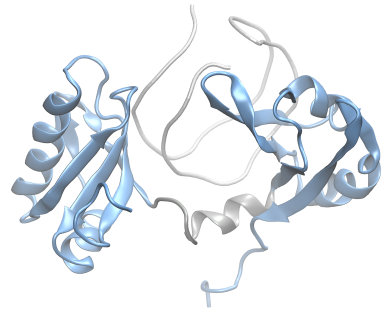  -217.78 |
| 9 | 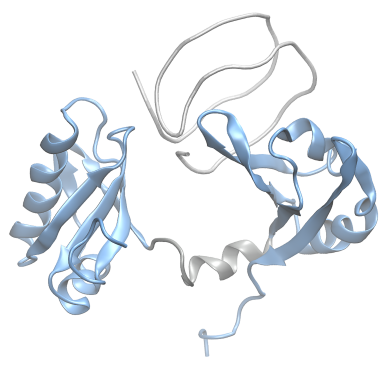  -211.82 | 10 | 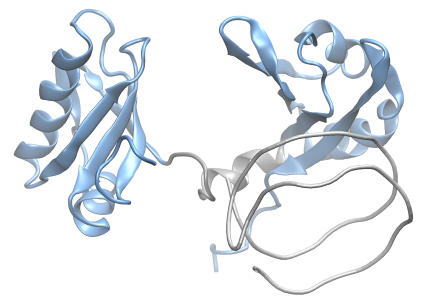  -211.43 |

**Supplementary Table S3.** Cluster and the superposition conformations of the 20 refined structures (PDB ID: 2KRR).

| cluster1  (1,2,3,6,7,8,13,18,19) | cluster2  (4, 14, 15, 20) | cluster3  (9, 11, 16) | cluster4  (10, 12) | cluster5  (5,17) |
| --- | --- | --- | --- | --- |
| 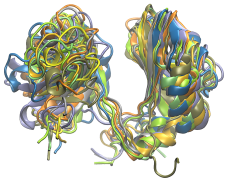 | 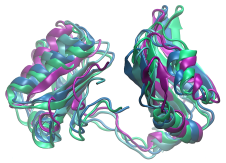 | 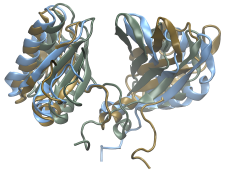 | 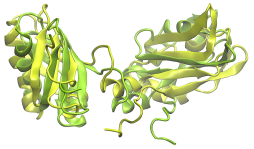 | 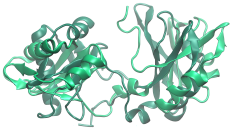 |

**Supplementary Table S4**. The top 10 predicted docking modes using model 4 of protein and AS1411 as input files of HDOCK (Yan, Tao et al. 2020). The proteins are shown in green NewCartoon mode and GQs are represented in silver Tube mode. The minimum energy from docking is shown under each structure with a unit of kcal/mol.

|  | structure |  | structure |
| --- | --- | --- | --- |
| 1 | 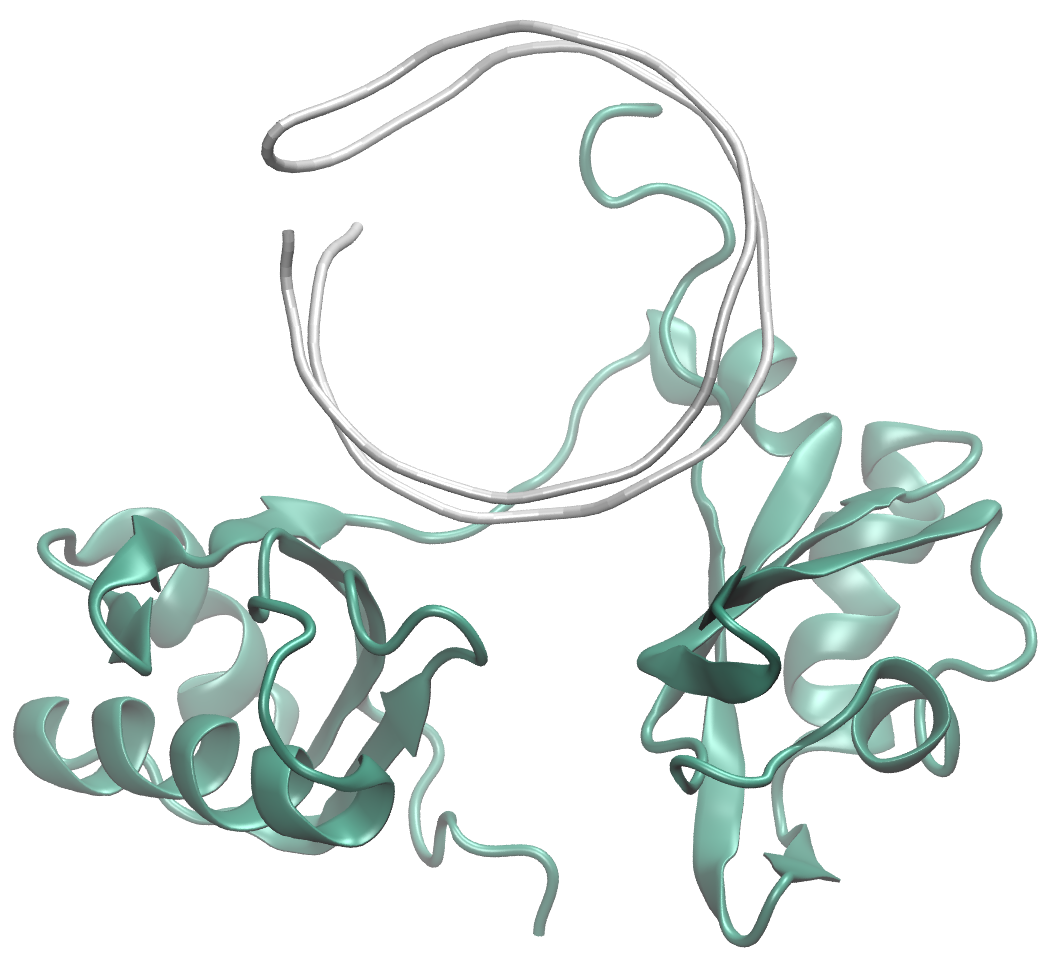  -232.48 | 2 | 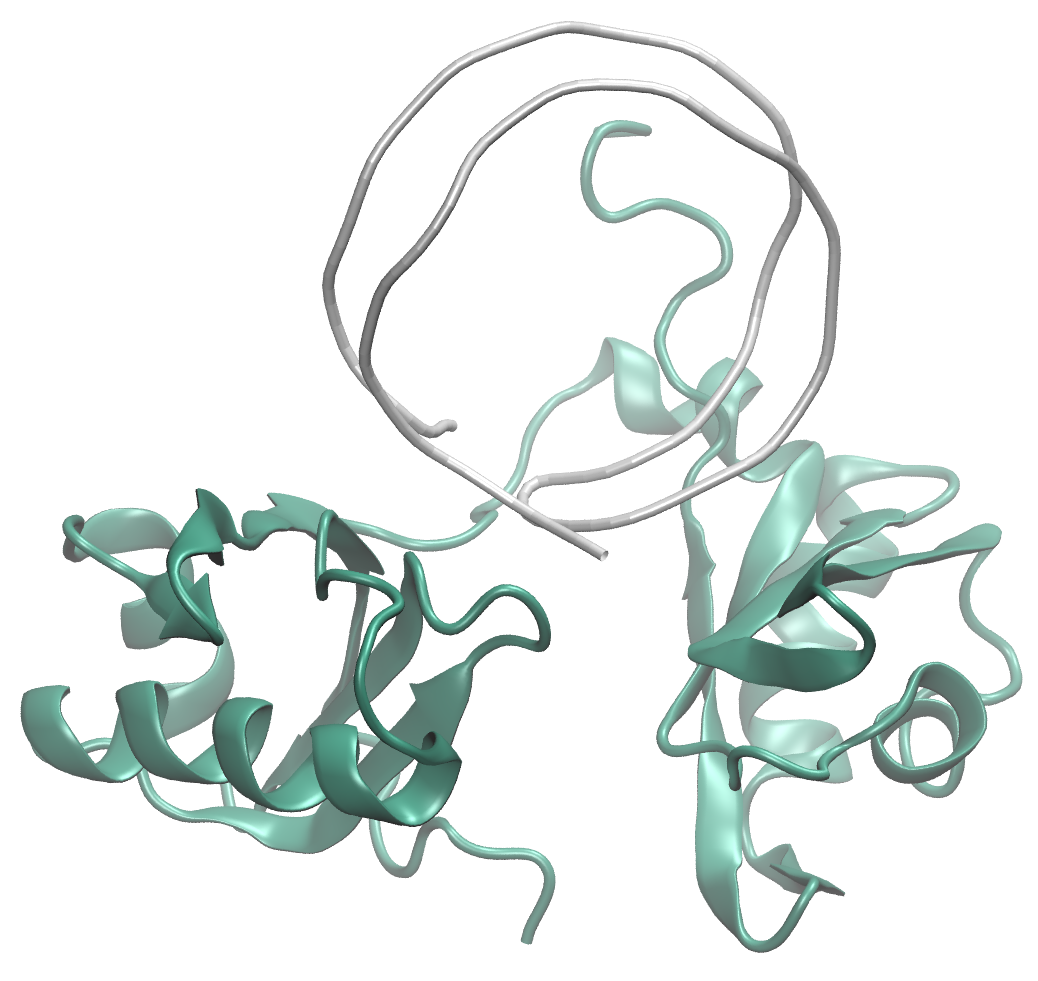  -231.63 |
| 3 | 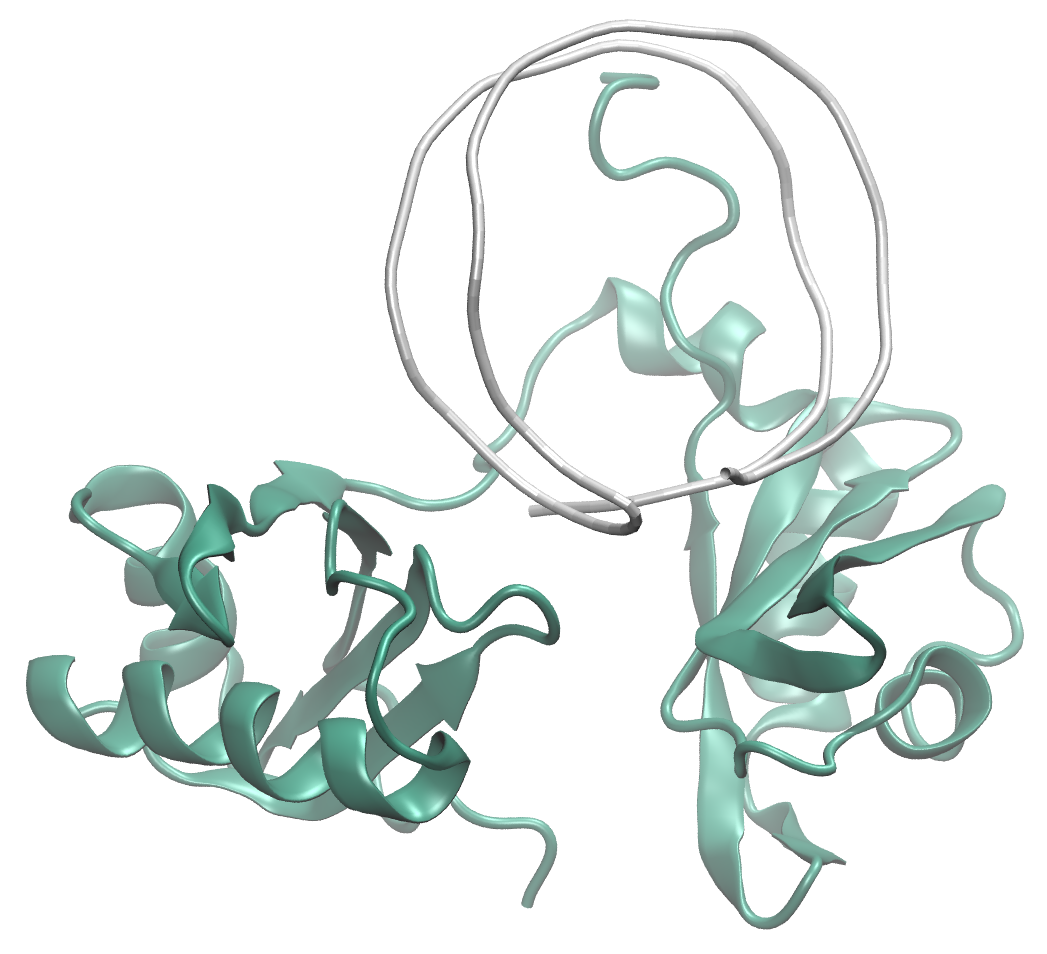  -229.03 | 4 | 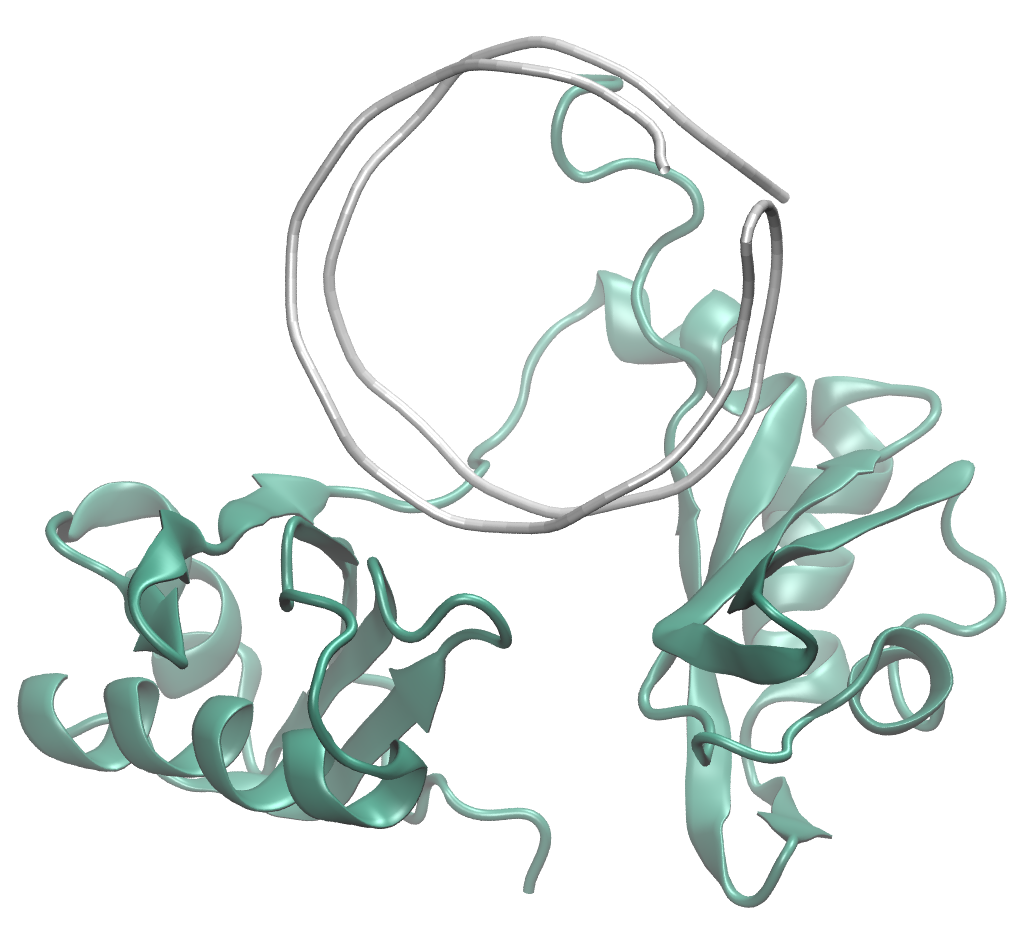  -228.19 |
| 5 | 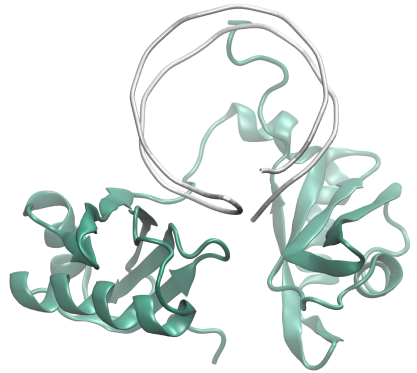  -226.23 | 6 | 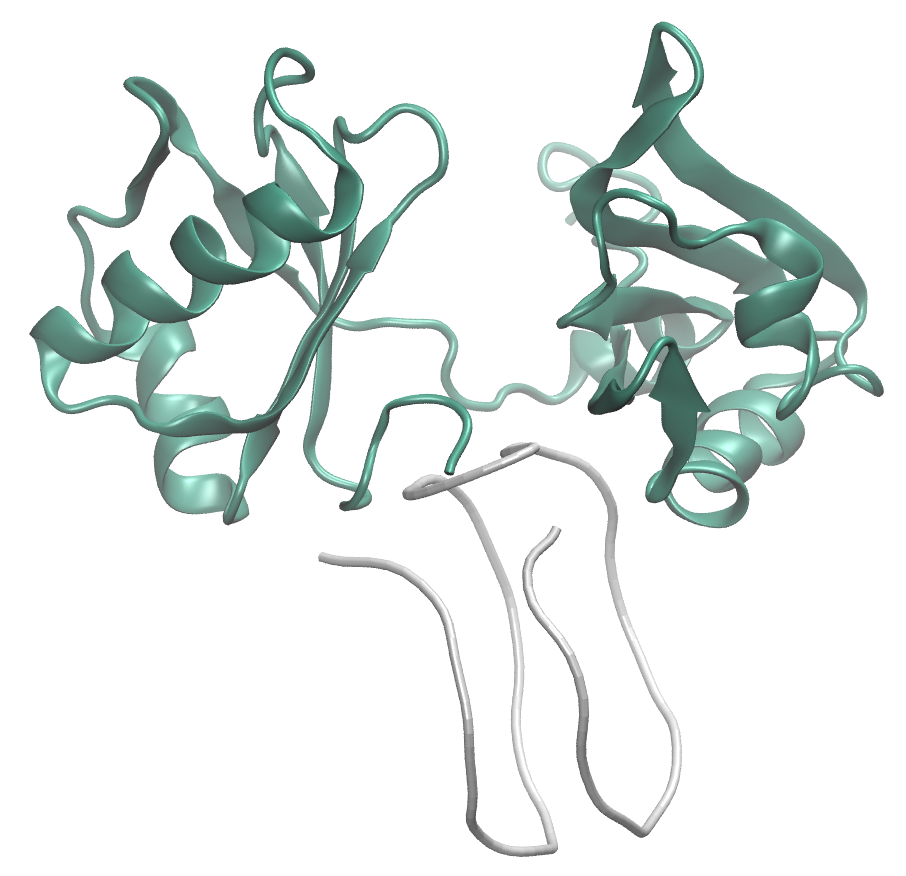  -215.68 |
| 7 | 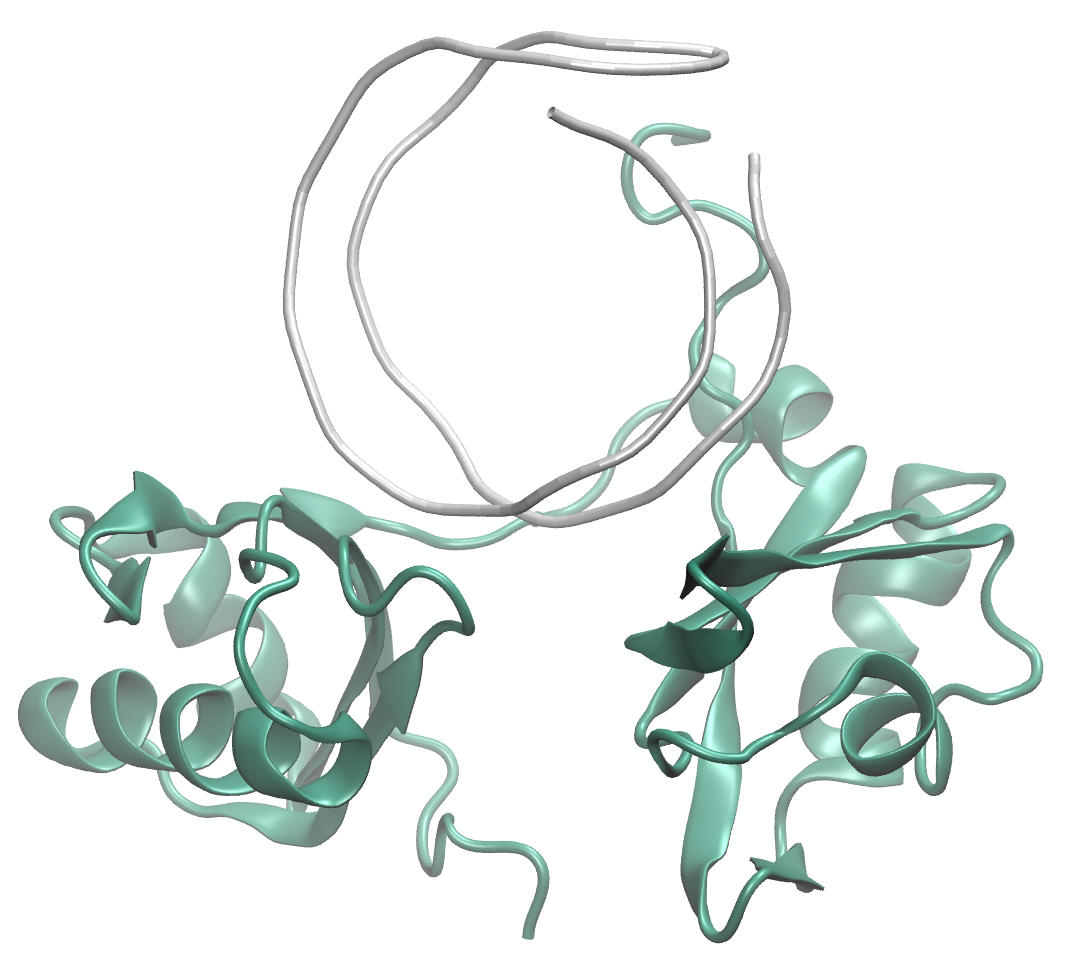  -215.25 | 8 | 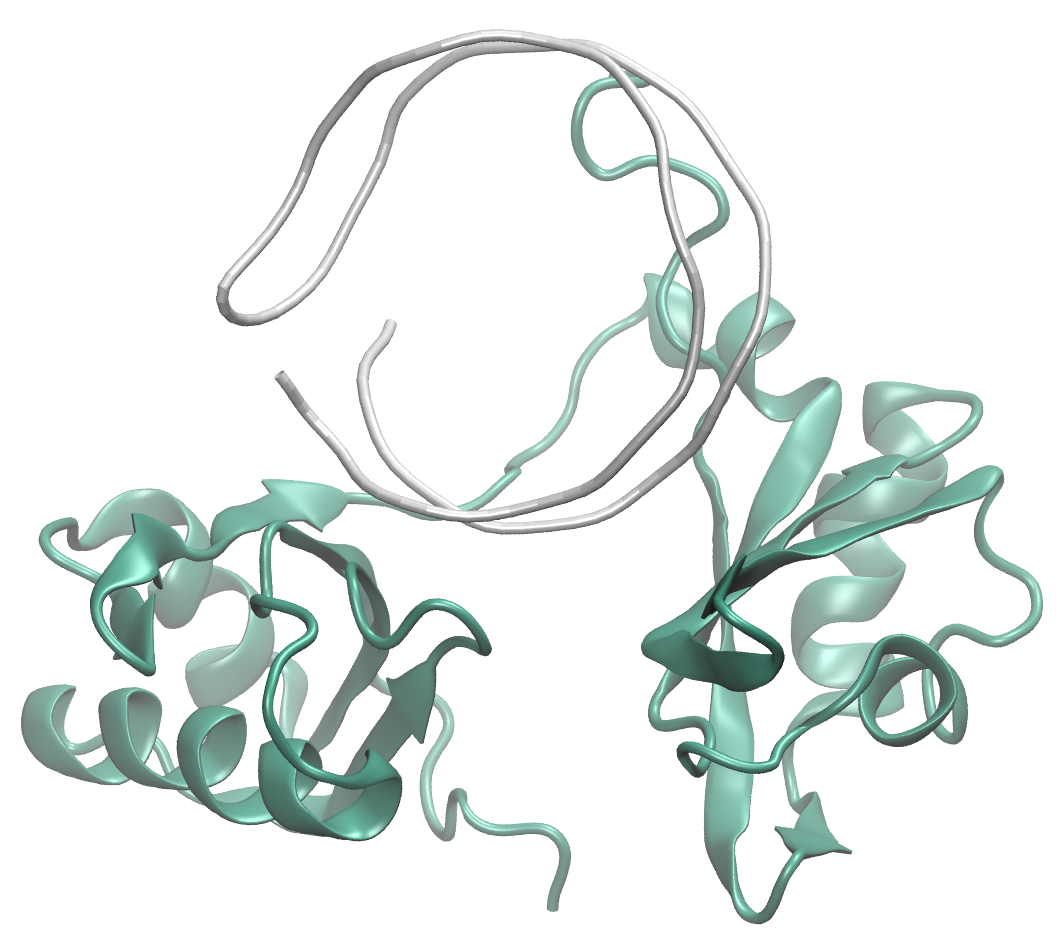  -213.21 |
| 9 | 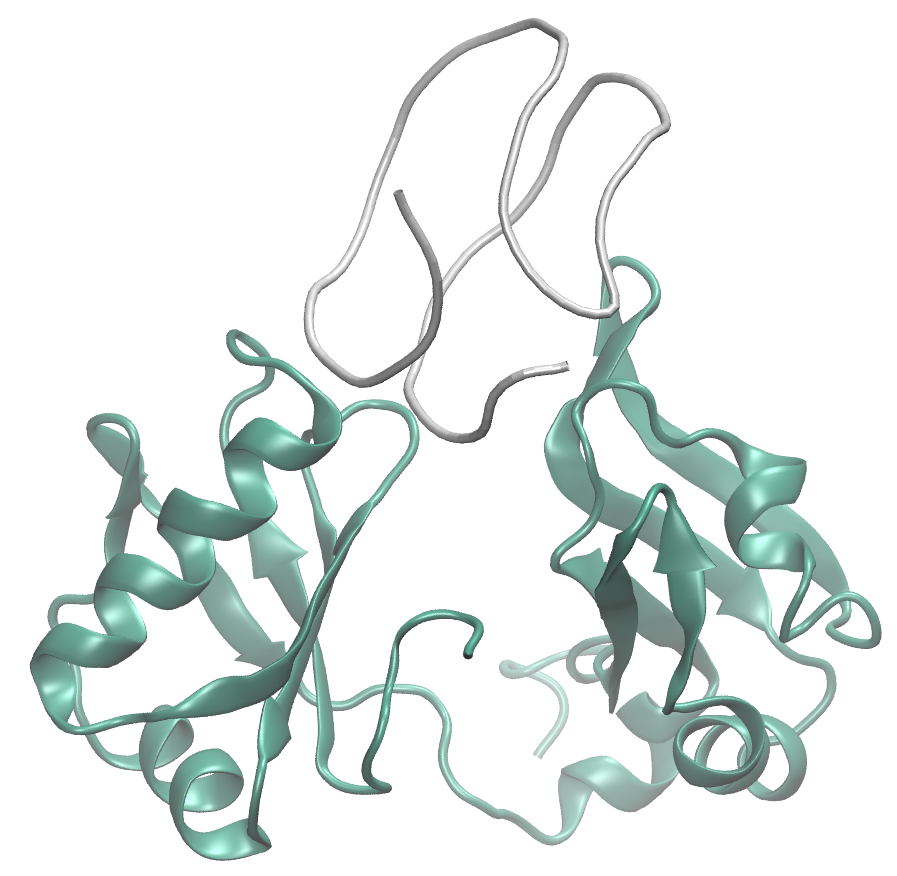  -211.34 | 10 | 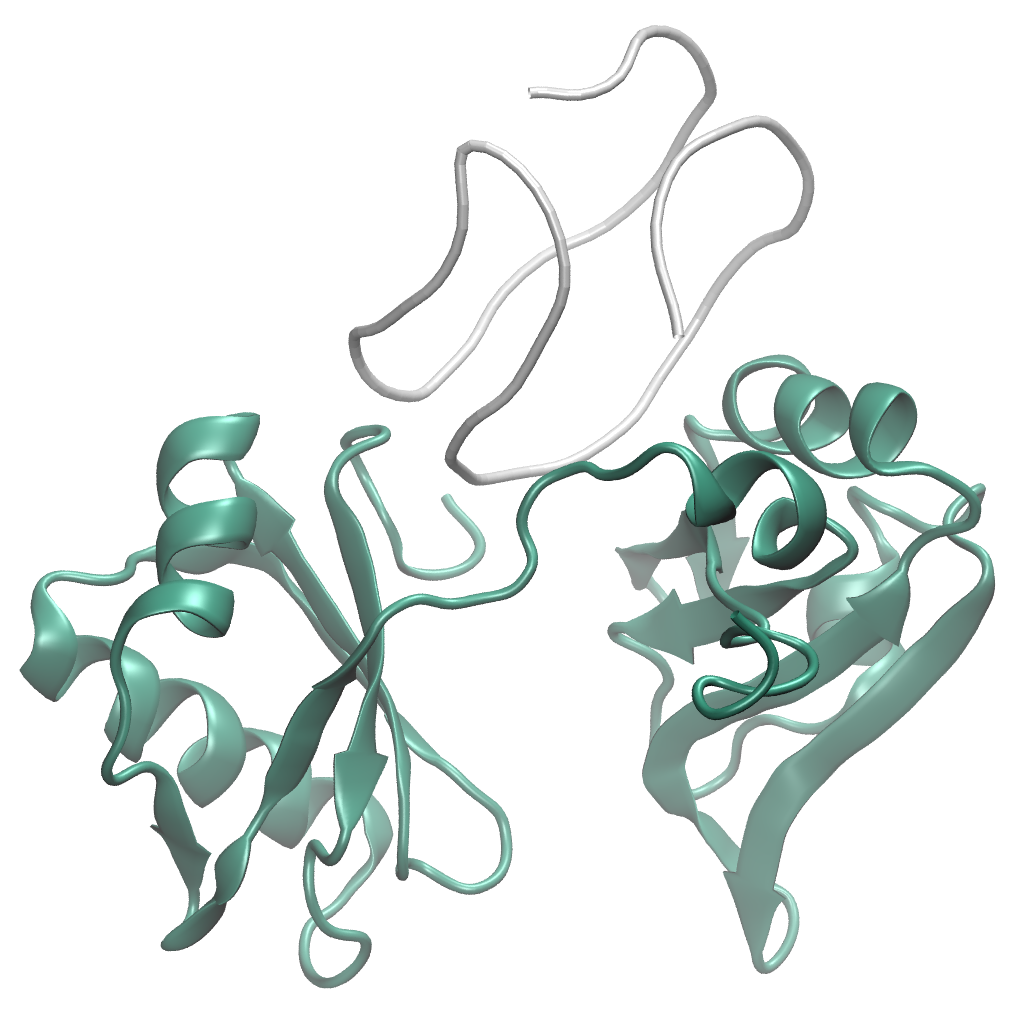  -211.18 |
